# Achieving High Coverage of H1N1 Influenza Vaccine in an Ethnically Diverse Obstetric Population: Success of a Multifaceted Approach

**DOI:** 10.1155/2011/746214

**Published:** 2011-06-26

**Authors:** Kara K. Hoppe, Linda O. Eckert

**Affiliations:** Department of Obstetrics and Gynecology, Harborview Medical Center, University of Washington, 1959 NE Pacific Avenue, P.O. Box 356460, Seattle, WA 98104-2499, USA

## Abstract

*Objective*. To report on a multifaceted approach to increase uptake of the H1N1 vaccine in our ethnically diverse obstetrical population. *Methods*. A review of our obstetric clinic vaccine registry and the approaches used to increase vaccine uptake. We created a real-time vaccine registry, educated patients in their own language via educational videos and use of cultural case workers, facilitated patient appointments and transportation, educated staff, and used other interventions to enhance immunization uptake. *Results*. Within the first month of H1N1 availability, we vaccinated 120 of our total 157 obstetrics patients. Our overall coverage rate was 76% (number vaccinated/total number eligible.) Of the enrolled patients, the vaccine acceptance rates were similar in our English (59 (78%) of 76) versus non-English (59 (75%) of 79) speaking patients. *Conclusions*. High vaccine coverage is possible in an ethnically diverse, highly immigrant obstetrics population.

## 1. Introduction

Pregnant women are known to be at increased risk for poor maternal and pregnancy outcomes with influenza [1] and are designated as a “high-risk” or priority population for receiving the influenza vaccine. Both the Advisory Committee on Immunization and Practices (ACIP) for the Center for Disease Control and Prevention and the American College of Obstetrics and Gynecology recommend immunization of all women who are pregnant during flu season in any trimester. Despite inclusion of seasonal influenza vaccine as an essential element of prenatal care [2], coverage rates have remained low and, in 2008-2009, the seasonal influenza vaccination rate in pregnant women was reported to be 11.3% [3]. 

A paucity of data exists as to the reasons for low vaccine coverage in an obstetric population. Many obstetric health care workers lack knowledge regarding the safety and clinical importance of influenza vaccine for pregnant women [4–6]. Studies are few addressing the relationship of obstetric patient population characteristics and immunization acceptance, and no data has addressed immunization acceptance in an immigrant obstetric population.

In 2009, a novel influenza A (H1N1) virus of swine origin caused human infection and acute respiratory illness in Mexico [1]. Rapid dissemination occurred and a pandemic was declared by the World Health Organization on June 11, 2009. Consistent with prior influenza pandemics, pregnant women suffered disproportionally. In the United States, pregnant women represent about 1 to 2% of the population but accounted for up to 7 to 10% of the hospitalized patients, 6 to 9% of the ICU patients, and 6 to 10% of those patients who died [1]. The quoted risk for hospitalization in pregnancy is increased by a factor of 4 to 7 as compared with age-matched nonpregnant women, with the highest risk in the third trimester [1]. Once the H1N1 influenza vaccine became available, CDC and ACOG strongly advised H1N1 vaccination for all pregnant women. 

Our clinic serves an ethnically diverse obstetric population. Many of our patients are recent immigrants unfamiliar with Western medicine and do not speak English. Because little data is published about immunization interventions in the population our clinic serves, we sought to analyze our experience. The purpose of this paper is to document a multifactorial highly successful influenza immunization program in our largely immigrant and ethnically diverse obstetric population.

## 2. Methods

### 2.1. Study Setting

The Harborview Medical Center (HMC) is located in Seattle, Wash, and is one clinical site for the University of Washington School of Medicine and serves an ethnically diverse population. The HMC Women's Clinic has 9 residents, 6 attendings, and one mid-level practitioner who provided obstetric care for 228 pregnant women in 2009. The HMC clinic utilizes certified medical interpreters available in person or by phone for patient visits. Additionally, the clinic offers cultural case workers who facilitate the integration of recent immigrants into a new medical system. Each pregnant patient is seen or offered an appointment with a social worker at least once during her pregnancy. 

### 2.2. Study Design

This is a retrospective study of H1N1 vaccine coverage achieved in the first month of vaccine availability in 157 pregnant women. All pregnant patients enrolled in our clinic at the time the vaccine became available on October 21st, 2009, were included. Using a standardized data form we collected data regarding age, ethnic background, spoken language, acceptance or refusal of the H1N1 vaccine, whether patients required clinic or hospital evaluations for influenza symptoms, and confirmed cases of H1N1. We linked clinic records to hospital records to determine patient outcomes and influenza hospitalization and influenza culture results. Our data was analyzed using Stata, and this study was approved by IRB no. 38805 at the University of Washington.

### 2.3. Interventions

Prior to vaccine availability we instituted educational sessions for team members in our obstetrics clinic. We presented data regarding the increased risk of H1N1 in pregnant women, recommendations for the influenza vaccine, and safety record of the vaccine. This included staff at our front desk, medical assistants, nurses, and social workers. Medical interpreters were also invited to attend. We planned future obstetrical visits to occur within two weeks of the anticipated vaccine release. Once the vaccine became available, patients were contacted personally in their own language to encourage clinic attendance and immunization, and those at or beyond 36 weeks gestation were offered taxi transportation. 

We placed an influenza prevention video made by our international medicine clinic in the waiting room that played continuously in 9 languages to increase the awareness of the influenza virus. It specifically discussed the importance of preventative measures such as receiving the immunization, symptoms, and receiving testing if our patients became symptomatic. Written literature was provided to the patients considering or receiving the vaccine. Patients were provided “flu packs” that contained masks, a digital thermometer, and hand antiseptic. 

**Figure 1:**
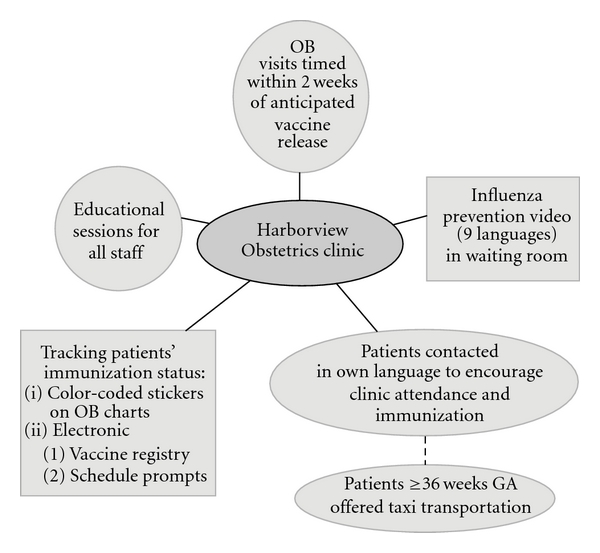
Clinic interventions used to increase H1N1 vaccine coverage.

We used dated H1N1 vaccine acceptance or refusal stickers on the front of our obstetric patient charts to prompt providers on the status of the patient. We created standing orders for the H1N1 vaccine administration that were placed in each chart to facilitate immunization at the time of the scheduled visit. An electronic vaccine registry was created by the nursing staff and updated daily, which allowed the clinic to easily identify the patients who had not yet received their vaccine. Because we had “real-time” information on vaccine administration, we were able to create electronic schedule prompts to track the patient's immunization status (Figure 1).

## 3. Results

Within the first month of vaccine availability, we immunized 120 (76%) of 157 enrolled obstetrical patients. Of the remaining patients, 17 declined, 5 delivered before the release of the vaccine, 2 contracted H1N1, 9 were lost to care and unreachable, and 9 planned to receive the vaccine at their next appointment. Of the 9 who planned to receive the vaccine at their next visit, 3 actually received it at their next prenatal visit, one patient delivered at an outside hospital and received it when they returned for their postpartum visit, and five did not get the vaccine upon return. No adverse events were documented in any of the patients who received the H1N1 vaccine. We had three confirmed cases of H1N1 in our obstetric population. One of the three patients received the H1N1 vaccine at 7-week gestation and contracted H1N1 7 days later. Of the two who did not receive the vaccine, one contracted H1N1 8 days after H1N1 vaccine availability. She had received the seasonal vaccine as this was available in our clinic prior to the H1N1 vaccine. The second contracted H1N1 at 15-week gestational age, which was 12 days after the vaccine availability. 

Demographic data for our study population are presented in Table 1. There was not a statistical difference in maternal age or gestational age and vaccine acceptance. The ethnic populations of the HMC obstetric patients who were included in this research analysis were 24.6% African American, 45.5% West/East African, 5.8% Pacific Islander/Asian, 1.3% Native American, 12.3% Caucasian, and 10.4% Hispanic. The acceptance rates across the different ethnic populations were 27 (71%) of 38 African American, 52 (74%) of 70 West/East African, 7 (78%) of 9 Pacific Islander/Asian, 2 (100%) of 2 Native American, 17 (90%) of 19 Caucasian, and 13 (81%) of 16 Hispanic. There was no difference between the acceptance rate across the different ethnic populations (*P* = .634). 

In a typical month 46% of our visits are interpreted visits. Vaccine acceptance was 59 (78%) of 76 in our English speaking versus 59 (75%) of 79 in our non-English speaking patients. We did not find a difference among the acceptance rate of the H1N1 vaccine in this analysis (*P* = .667) (Table 1).

## 4. Conclusions

Our overall coverage rate of 76% (number vaccinated/total number eligible) compares favorably to the nationwide 38% coverage rate reported initially by the CDC and the 46.6% reported more recently from 10 states using the Pregnancy Risk Assessment Monitoring System (PRAMS) [7, 8]. Despite inclusion of seasonal influenza vaccine as an essential element of prenatal care [2], a reassuring safety profile in all trimesters of pregnancy [9, 10], and documented benefit to newborns and infants whose mothers receive the influenza vaccine while pregnant [2, 11–13], coverage rates of pregnant women with seasonal influenza vaccine have been minimal [3]. Cognizant of this historically low coverage rate, we began planning weeks in anticipation of the vaccine to institute multiple interventions. We were able to document high H1N1 immunization acceptability and coverage levels among our ethnically diverse obstetrics population. 

Previous studies suggest that both obstetric and nonobstetric healthcare workers lack confidence in the safety of the influenza vaccine in pregnancy [6, 14, 15]. Additionally, our office colleagues may be unaware of the distinct benefit influenza vaccination offers mothers and infants. This led us to proactively institute educational sessions for all of our staff prior to availability of the vaccine. We offered a straightforward message stressing 3 of the strong reasons for immunizing all pregnant women with influenza vaccine: pregnant women are more likely to acquire influenza after an exposure, they become more sick when they contract influenza, and immunizing the mother during pregnancy decreases hospitalizations and respiratory illnesses in their infants. The increased understanding and awareness among our staff of the importance and safety of influenza vaccination in pregnant women encouraged a successful team approach and is likely a key factor in achieving a high rate of vaccine acceptance and administration.

The use of a vaccine registry has not been published in an obstetric population but is used commonly in pediatric populations and more recently in adult populations [16, 17]. We developed an electronic vaccine registry that was updated daily. This allowed us to target patients who had not received the vaccine, anticipate their next appointment, and ensure all patients were offered the vaccine in an expedited fashion. In addition we had a sticker placed on each paper chart to identify the exact date the vaccine was administered or declined. With these tools, we could efficiently track and communicate information regarding our patients' vaccine status. Automatic prompts are also available within many electronic medical record systems. While these immediate visual cues for the staff and provider appeared to have excellent utility in our clinic, research is needed to compare the impact of various “triggers” on immunization rates.

Our clinic serves a multiethnic population. We searched for published data on immunization programs in such a patient population but found scant information was available to assist either providers or immunization program managers in obstetric clinics who serve a multiethnic population. The CDC has demonstrated disparities in vaccination coverage among Hispanics and blacks [18]. Proposed barriers to vaccination in these populations include ability to access healthcare, language barriers, and fear of being able to provide proof of legal status to obtain vaccination [18]. In our diverse ethnic group, we found no difference in vaccine acceptance across all our ethnic populations between English and non-English speaking patients, or between country/culture of origin. We have excellent interpreter services and were able to provide our patients with spoken and written information in many languages. This ability to communicate effectively with our non-English speaking patients undoubtedly contributed to the equivalent vaccine coverage despite spoken language.

Increased media attention regarding H1N1 in 2009 both encouraged and discouraged the use of immunization. We are unable to separate out the impact of this strong media attention versus our clinic efforts in achieving high coverage. Before the vaccine was available, data confirming the increased risk of H1N1 in pregnant women was published [1]. We educated patients using standard safety messages and emphasized the increased threat to pregnant patients with the current season of H1N1. With some patients, lengthy discussions of safety and testing of the vaccine were needed to address the media reports of adverse events attributed to both vaccine uses in general and the H1N1 vaccine in particular. 

This paper has several limitations. Our success is reported from a single clinic which is ethnically diverse and has the support of interpreter services and cultural case workers which may limit the ability to generalize this study to other clinics. We acknowledge that we instituted multiple interventions simultaneously. While we may hypothesize that reaching out to our patients with culture case workers in their own language or educating the staff in the clinic may be the most important variables, we are not able to delineate the relative importance of each factor on vaccination rates during the initial H1N1outbreak. Additionally, the hospital allocated additional resources to our clinic including provision of influenza care packages, as well as increased staffing to answer phone calls and triage patients. These additional resources may have provided an improvement in vaccine acceptability which is not sustainable. We have ongoing monitoring to compare 2009 coverage rates with current influenza immunization uptake now that we are not in a nonpandemic setting. Lastly, we have no comparator clinic where these interventions were not instituted.

Despite these limitations, we believe the success of this multifaceted approach, with interventions designed, in part, to target our highly diverse, immigrant obstetric population, may provide a useful model for other clinics. Additionally, we anticipate that this study may inform future research trials designed to improve vaccine coverage in this typically undervaccinated population.

## Figures and Tables

**Table 1:** Demographic and outcome characteristics of vaccinated versus unvaccinated HMC obstetric patients.

Variable	All patients *n* = 157	Vaccine acceptance *n* = 120 (76%)	Vaccine declination *n* = 37 (24%)	*P* value
Maternal age mean*	27.8	27.5 ± 6.1	28.7 ± 5.5	.346^+^
Gestational age mean*	23.6	23.9 ± 9.8	26.6 ±12.8	.195^+^
Ethnic populations** *N* = 154				.634^++^
African American	38	27 (71%)	11 (29%)	
West/East African	70	52 (74%)	18 (26%)	
Pacific Islander/Asian	9	7 (78%)	2 (22%)	
Native American	2	2 (100%)	0 (0%)	
Caucasian	19	17 (89%)	2 (11%)	
Hispanic	16	13 (81%)	3 (19%)	
Spoken language*** *N* = 155				
English	76	59 (77%)	17 (23%)	.667^++^
Non-English	79	59 (75%)	20 (25%)	

^+^ *T-*test.

^++^Chi square.

*n = 153 due to missing information regarding maternal and gestational age.

**n = 154 due to missing information regarding ethnic background.

***n = 155 due to missing information regarding spoken language.
